# Immune Cell-Epithelial/Mesenchymal Interaction Contributing to Allergic Airway Inflammation Associated Pathology

**DOI:** 10.3389/fimmu.2019.00570

**Published:** 2019-03-26

**Authors:** Kiyoshi Hirahara, Kenta Shinoda, Yuki Morimoto, Masahiro Kiuchi, Ami Aoki, Jin Kumagai, Kota Kokubo, Toshinori Nakayama

**Affiliations:** ^1^Department of Immunology, Graduate School of Medicine, Chiba University, Chiba, Japan; ^2^AMED-PRIME, AMED, Chiba, Japan; ^3^Laboratory of Genome Integrity, National Institutes of Health, Bethesda, MD, United States; ^4^AMED-CREST, AMED, Chiba, Japan

**Keywords:** iBALT, pathogenic Th2 (Tpath2) cells, fibrosis, Amphiregulin (AREG), osteopontin (OPN, Spp1), inflammatory eosinophils

## Abstract

The primary function of the lung is efficient gas exchange between alveolar air and alveolar capillary blood. At the same time, the lung protects the host from continuous invasion of harmful viruses and bacteria by developing unique epithelial barrier systems. Thus, the lung has a complex architecture comprising a mixture of various types of cells including epithelial cells, mesenchymal cells, and immune cells. Recent studies have revealed that Interleukin (IL-)33, a member of the IL-1 family of cytokines, is a key environmental cytokine that is derived from epithelial cells and induces type 2 inflammation in the barrier organs, including the lung. IL-33 induces allergic diseases, such as asthma, through the activation of various immune cells that express an IL-33 receptor, ST2, including ST2^+^ memory (CD62L^low^CD44^hi^) CD4^+^ T cells. ST2^+^ memory CD4^+^ T cells have the capacity to produce high levels of IL-5 and Amphiregulin and are involved in the pathology of asthma. ST2^+^ memory CD4^+^ T cells are maintained by IL-7- and IL-33-produced lymphatic endothelial cells within inducible bronchus-associated lymphoid tissue (iBALT) around the bronchioles during chronic lung inflammation. In this review, we will discuss the impact of these immune cells-epithelial/mesenchymal interaction on shaping the pathology of chronic allergic inflammation. A better understanding of pathogenic roles of the cellular and molecular interaction between immune cells and non-immune cells is crucial for the development of new therapeutic strategies for intractable allergic diseases.

## Mucosal Inflammation and iBALT

### Induction of Inducible Bronchus-Associated Lymphoid Tissue (iBALT)

Pulmonary immunity is the first line of defense against pathogenic microorganisms and a unique local immune system that is distinct from systemic immunity. Following pulmonary inflammation, highly organized lymphoid structures known as iBALT are induced adjacent to the bronchus and next to a vein and artery. Similar to the secondary lymphoid organs (SLOs), iBALT consists of separated B cell areas with follicular dendritic cells (FDCs), where germinal centers can develop, and T cell areas harboring dendritic cells (DCs) as well as high endothelial venules (HEVs). It has been proposed that iBALT serves as an inducible lymphoid tissue for pulmonary immune responses. Since iBALT is not formed in a pre-programed way and induced by various types of pulmonary inflammation, it has been recognized as the tertiary lymphoid organs. The induction of iBALT depends on the type of antigens and the magnitude of immune responses, and its cell number and size depend on the duration of antigenic exposure (1, 2). iBALT persists in the lung for months after inflammation resolved and plays an important role in the protection against re-invasion of pathogens.

A previous study examined the adaptive immune response of mice that forms iBALT but with the complete absence of SLOs in the body following inoculation with influenza virus (3). Surprisingly, mice without SLOs are still able to respond and clear a challenge infection, and influenza-specific virus-neutralizing antibodies are generated and maintained by the presence of iBALT. Thus, iBALT may be sufficient to protect against pathogens. It is known that modified vaccinia virus Ankara (MVA)-induced iBALT is able to become a general priming site against various pulmonary antigens [i.e., not only against the iBALT-initiating antigen but also against unrelated antigens (4)].

Given that the structure of iBALT is similar to that of conventional SLOs, there are many similarities in the molecular mechanisms underlying the development of these lymphoid organs. Lymphotoxin (LT)-alpha-deficient mice have defects in the development of LNs or Peyer's patches because of the reduction of homeostatic chemokines (CCL19, CCL21, and CXCL13) production by specialized stromal cells that express LTβR and tumor necrosis factor receptor 1 (TNFR1) (5–8). These homeostatic chemokines attract lymphocytes and promote lymphoid organ structure development. The development of iBALT also requires LT signaling, which leads to homeostatic chemokine production during chronic inflammation. CXCL13 and CCL19 have been shown to be controlled by LT-dependent mechanisms in a smoke-exposed chronic obstructive pulmonary disease (COPD) model (8). In addition, *plt/plt* mice, which lack CCL19 and CCL21, and *Cxcl13*^−/−^ mice show attenuated iBALT formation after influenza infection. Furthermore, *Cxcl13*^−/−^*plt/plt* mice fail to form detectable iBALT formations, suggesting that the combination of homeostatic chemokines is crucial for iBALT formation (9).

Several differential mechanisms exist in the development of iBALT and conventional lymphoid organs. For example, the transcriptional regulators inhibitor of DNA binding 2 (Id2) and RAR-related orphan receptor gamma t (RORγt), which are essential for the differentiation of lymphoid tissue inducer cells and organogenesis of peripheral lymph nodes and Peyer's patches, are dispensable for iBALT organogenesis (10–13). Formation of both iBALT and omentum milky spots were associated with CXCL13, but this was dispensable for tear-duct associated lymphoid tissue (TALT) (11, 14, 15). Interestingly, it has been reported that the expression of CXCL13 and CCL19 is controlled by IL-17-producing Th17 cells and is required for iBALT development, whereas LT signaling is required in the maintenance of iBALT formation (11). Furthermore, iBALT organogenesis has been shown to be induced by CD11b^+^CD11c^+^ DCs, which produce homeostatic chemokines and LT in response to influenza or vaccinia virus infection (4, 16). In addition, CCR7^+^ Treg cells have been reported to suppress the development of iBALT (17).

Specialized stromal cells and lymphatic vessels are found in iBALT and play a critical role in the regulation of iBALT structure and various immune function. For example, in the B cell area of iBALT, there is a network of CXCL13-expressing FDCs as well as CXCL12-producing follicular stromal cells (18). In contrast to CXCL12 and CXCL13, CCL21 is found on HEVs in T cell areas but not in B cell follicles. Similar to the secondary lymphoid organs, lymphatics are developed in the T cell area and surrounding B cell follicle in the iBALT and likely facilitate the uptake of antigens (19–21). However, it is difficult to determine histologically whether these lymphatics are afferent and carry antigens and lymphocytes from distal portions of the lung toward iBALT or whether efferent and carry lymphocytes from iBALT and return them to the general circulation.

A previous report showed by the live imaging of *ex vivo* lung tissue that labeled antigen-pulsed DCs can migrate from airways into iBALT and prime T cell responses (4), suggesting that DC migration to iBALT is an important mechanism by which antigens are acquired for local immune responses. Epithelial antigen-transporting M cells are detected in iBALT of some species (22–24) and are also involved in the uptake of antigens in iBALT.

**Figure 1:**
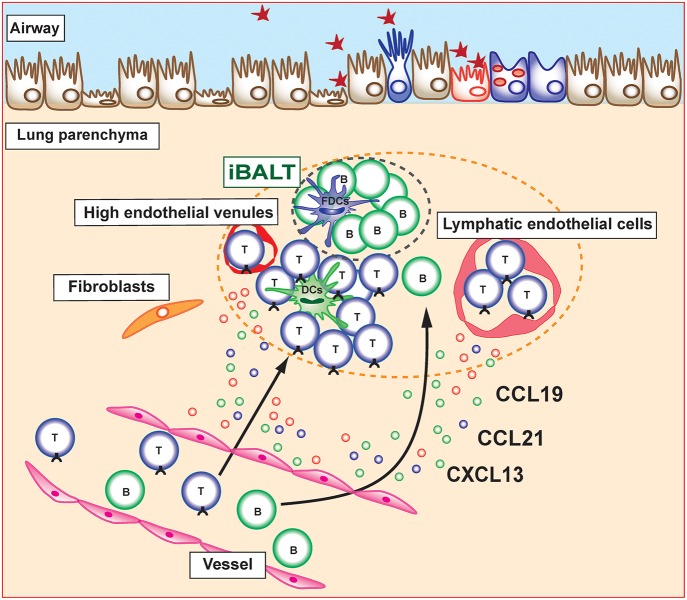
Induction and maintenance of inducible bronchus-associated lymphoid tissue (iBALT). Inducible bronchus-associated lymphoid tissue (iBALT) is formed in the lung during chronic inflammation and consist of various type of immune cells, including T cells, B cells, dendritic cells (DCs), and follicular dendritic cells (FDCs). CCL19, CCL21, and CXCL13 are key chemokines for the induction and maintenance of iBALT structure. Stromal cells including fibroblasts, vascular endothelial cells, and lymphatic endothelial cells are the major producers of these chemokines in the lung parenchyma.

Several mechanisms underlying the recruitment of immune cells into already developed iBALT structure have been reported. The migration of naive T cells to lymph nodes via HEVs was shown to be mediated by adhesion molecules, such as integrins or selectins, as well as by chemokine receptors (25). Similarly, the migration of lymphocytes into iBALT is mediated by adhesion molecules and chemokines. Lymphocyte homing is controlled by L-selectin/Peripheral node addressin (PNAd), α4β1 integrin/Vascular cell adhesion molecule 1 (VCAM1), and the lymphocyte function-associated antigen 1 (LFA-1) adhesion pathway, whereas α4β7 integrin and Mucosal vascular addressin cell adhesion molecule (MAdCAM) are not involved in the spontaneous iBALT formation of autoimmune-prone NOD mice (26). Similar mechanisms have been found in iBALT associated with lung carcinoma in which B cells and some T cells expressed L-selectin and α4 integrin, suggesting that these adhesion molecules are involved in the migration of lymphocytes into human iBALT (27). Relevance of chemokine receptors on iBALT-infiltrating lymphocytes has been shown to be involved in trafficking of lymphocytes toward and within the lung (28, 29). In addition, multiple chemokines (CCL17, CCL19, CCL21, CCL22, CXCL13, and IL-16) are expressed in tumor-induced iBALT in lung cancer patients and their respective receptors (CCR7, CCR4, and CXCR5) are expressed on T cells within iBALT. Interestingly, we previously reported that specialized Thy1^+^ lymphatic endothelial cells in iBALT have the ability to produce IL-7 as well as the T cell attractant chemokines CCL19 and CCL21 (30) (Figure 1). Furthermore, these Thy1^+^IL-7^+^ lymphatic endothelial cells also produce IL-33, an inflammatory cytokine involved in the pathogenesis of allergic immune responses. It is expected that there are other unknown stromal cells that produce lymphocyte attractant chemokines within iBALT and regulate the trafficking of lymphocytes into iBALT. Further detailed analyses focusing on their characterization are required.

For the analysis of iBALT, various methods associated with iBALT formation have been investigated. BALT is not detectable in the lungs of naïve mice, but it has been reported that spontaneous iBALT formation is found in chemokine receptor CCR7-deficient mice (17). iBALT can also be induced by the intranasal administration of viruses, including influenza virus, poxvirus MVA, and murine herpes virus-68 (4, 20, 31). Furthermore, lipopolysaccharide (LPS) intranasal administration in newborn mice (11) or repetitive inhalations of heat-killed bacteria (18, 32) induce iBALT. iBALT formation has also been shown to be associated with chronic airway inflammation. Mouse models of chronic cigarette smoke-induced COPD model or severe antigen-induced experimental asthma model are also associated with iBALT formation in the lungs (33), and the iBALT areas increase with increased antigen exposure. The types of inflammation-inducing antigen appear to determine the cellular component that forms and is required for the maturation of iBALT (18). For instance, mice intranasally treated with poxvirus MVA develop highly organized iBALT with densely packed B cell follicles containing a network of FDCs with CXCL12-producing follicular stromal cells. In contrast, *Pseudomonas aeruginosa* treatment leads to iBALT without FDCs but still containing CXCL12-positive follicular stromal cells within B cell follicles. Thus, the type of antigen or magnitude of inflammation may affect the nature of iBALT formed in the lung.

Further detailed analyses of classification of iBALT are required to explore their distinct function in pulmonary immunity.

### Pathologic Roles of iBALT in Allergic Airway Inflammation: The Interaction of Pathogenic Th2 (Tpath2) Cells and Lymphatic Endothelial Cells in iBALT

In contrast to the protective role of iBALT in infectious diseases, iBALT structures are frequently detected in the lung tissue obtained from patients with asthma (2, 34) and are evident in mouse models of experimental asthma (30, 35, 36). It has been shown in clinical analyses that the cases with asthma tend to have larger and greater numbers of lymphoid cell aggregates in the lung than non-asthma cases, regardless of asthma severity (34). More recently, iBALT-like lymphoid structures were detected in a biopsy of the inner bronchial wall in mild asthma patients (37). Several mouse models for allergic airway inflammation have been shown to be accompanied by the formation of iBALT. We and others have found that the induction of severe allergic lung inflammation by transferring highly Th2-polarized respiratory allergen-specific TCR transgenic CD4 T cells followed by repeated intranasal challenges with allergen induced iBALT in the lung (30, 35). The overexpression of IL-5 in mice was reported to develop iBALT formation, although whether or not this response is controlled via a direct effect on B cells remains to be determined (38). A histological examination of the lung treated with anti-IL-4 and anti-IL-13 antibodies also revealed that IL-4 and IL-13 have the potential to induce iBALT formation (35). Thus, it appears that allergic lung inflammation facilitates the formation of iBALT, which is involved in the pathogenesis of asthma.

Accumulating evidence has shown that iBALT is the structure supporting the maintenance of immunological memory. In a mouse model of allergic lung inflammation, antigen-specific IgE-secreting plasma cells have been shown to reside adjacent to the germinal centers in iBALT (39). Similarly, patients with allergic bronchopulmonary aspergillosis also develop iBALT areas, some of which have allergen-specific IgE-expressing B cells in the germinal centers (40). Furthermore, we recently demonstrated that the lung-resident antigen-specific memory Th2 cells preferentially reside in iBALT and are sufficient to induce asthmatic symptoms (30). Similarly, it has also been shown that lung-resident memory Th2 cells are sufficient to induce airway hyperresponsiveness by experimental blockade of lymphocyte migration (41). Unique clinical evidences from the patients with lung transplantation contributed to prove the concept proposed from mice experiments (42). The recipients without chronic airway inflammation developed asthma after the transplantation of the asthmatic lung. In contrast, the recipient with chronic airway inflammation did not suffer from asthma after the non-asthmatic lung transplantation. These findings suggest that asthma is a local disease and iBALT formation is involved in it.

**Figure 2:**
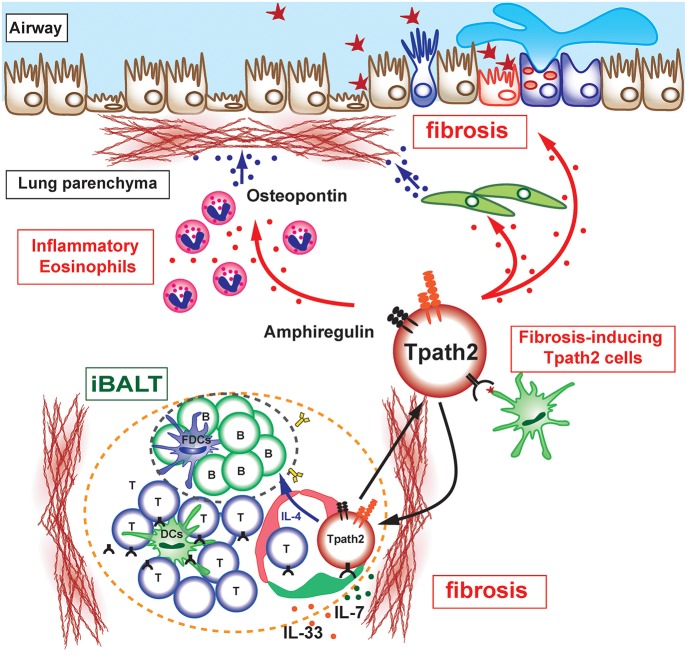
Pathogenic interaction between immune cells and epithelial/mesenchymal cells in allergic airway inflammation. The selective localization and survival of memory-type Tpath2 cells within iBALT are supported by IL-7^+^ lymphatic endothelial cells (LECs). These IL-7^+^ LECs are also IL-33^+^ and may therefore induce memory Th2 cells to be more pathogenic. A subpopulation of memory-type pathogenic Th2 (Tpath2) cells produces Amphiregulin, reprogramming eosinophils to the inflammatory state via the production of osteopontin and facilitating the fibrotic responses in the airway.

Memory-type pathogenic Th2 cells are the principal cell population responsible for the pathogenesis of chronic allergic inflammation and the rapid relapse of acute allergic inflammation upon allergen re-exposure in mice (36). The areas of iBALT may efficiently collect antigen-bearing DCs or macrophages via lymphatics or by other mechanisms, promoting their concentration in areas devoted to T and B cell priming and simultaneously depleting them from the rest of the lung. In fact, plasmacytoid DCs in patients with asthma or moderate COPD are found to be concentrated in iBALT areas of the lung (43) where they may promote the local differentiation of Tregs. The mechanisms that regulate the maintenance of lung-resident memory Th2 cells have been incompletely defined. Tissue-resident memory CD4 T cells have been considered to be actively transported to the SLOs in order to interact with IL-7-producing stromal cells (44). However, parabiosis experiments in which mouse pairs are surgically conjoined to create shared circulation systems provide direct evidence for the retention of memory CD4 T cells in lung tissues (45). We recently clarified that the maintenance of lung-resident antigen-specific memory Th2 cells is dependent on IL-7-producing lymphatic endothelial cells (LECs), which localize within iBALT (30). In fact, antigen-specific memory Th2 cells are maintained in an IL-7-dependent manner but with an antigen-independent mechanism. Furthermore, Th2 cells, which are not specific to iBALT-initiating antigen, are still maintained within the iBALT and strongly contribute to the pathology of local pulmonary inflammation. Notably, IL-7-producing LECs increase their number after lung inflammation with the increased formation of iBALT, consistent with the fact that robust lymphangiogenesis occurs in mouse lungs after pulmonary infection and the lymphatic network is restricted to regions of iBALT (46). Thus, it appears that allergic asthmatic responses can cause iBALT formation and modify the lung microenvironment to promote the survival of antigen-specific memory Th2 cells within iBALT. Interestingly, the analysis of IL-7-producing LECs reveals the unique characteristic of this cell population as a producer of IL-33. It has been shown that IL-33 directly instructs memory type pathogenic Th2 (Tpath2) cells to enhance IL-5 production and induces eosinophilic inflammation (47). Interestingly, the adoptive transfer of *Il-1rl1*^−/−^ Th2 cells, which have a defective expression of IL-33 receptor (ST2), can induce the formation of iBALT and thus can be maintained within the structure, but the ability to produce IL-4, IL-5, and IL-13 in *Il-1rl1*^−/−^ Th2 cells dramatically decreases (48). This result shows that the IL-33-ST2 signaling pathway is critical for ensuring that Tpath2 cells maintain the ability to secrete Th2 cytokines. Thus, IL-7- and IL-33-producing LECs may provide a niche for Tpath2 cells to maintain their survival and pathogenicity in the lung (Figure 2). These findings suggest that IL-7- and IL-33-producing LECs within iBALT contribute to the trafficking, activation, and survival of Tpath2 cells and have a potential regulatory function in chronic allergic airway inflammation.

In fact, ectopic lymphoid structures are often developed within upper-respiratory airways with inflammation. Eosinophilic chronic rhinosinusitis (ECRS) is a chronic upper-respiratory airway allergic disease characterized by massive eosinophilic infiltration and high IL-5 levels associated with the formation of recurrent nasal polyps (49). We detected memory Th (CD45RO^+^CD4^+^) cells along with antigen-presenting cells and IL-7 and IL-33-producing LECs in ectopic lymphoid tissues of patients with ECRS, similar to that found in iBALT in mice (30). These findings suggest that similar cellular and molecular mechanisms underlying the maintenance of immunological memory at ectopic lymphoid structures may operate in the upper- and lower-respiratory airway, which is involved in the pathogenesis of chronic airway inflammation.

## Pathogenesis of Tissue Fibrosis in Allergic Airway Inflammation

Memory Th2 cells are considered to be a key cell population in the pathogenesis of chronic inflammatory disorders including asthma. However, the specific subpopulations of memory Th cells and the factors that induce chronic airway inflammation, particularly fibrotic responses, remain unclear due to limited numbers of antigen-specific memory Th cells recoverable from inflammatory respiratory tissues. Several investigators, including us, have recently identified phenotypically and functionally distinct memory Th2 cell subsets that produce large amounts of IL-5 in addition to IL-4 and IL-13 (36, 50, 51). Amphiregulin, a ligand for epithelial growth factor receptor (EGFR), has crucial roles in inflammation, tissue repair and fibrotic responses (52). Amphiregulin has been reported to be produced by effector Th2 cells (53). We recently identified an Amphiregulin-producing subpopulation of memory Tpath2 cells that is critical for shaping the pathology of fibrosis during chronic allergic inflammation (54). We found that Amphiregulin is a crucial mediator in the induction of fibrotic responses in chronic allergic inflammation. Amphiregulin-EGF receptor (EGFR)-mediated signaling promotes the reprogramming of eosinophils to an inflammatory state with enhanced production of osteopontin, a major component of the non-collagenous ECM in fibrotic tissues and a contributor to the pathogenesis of fibrosis (Figure 2). Amphiregulin also stimulates lung epithelial cells to undergo proliferation and enhanced mucin production (53, 55) and promotes the proliferation of lung fibroblasts (56). Moreover, Amphiregulin by macrophage induces TGF-β activation and the trans-differentiation of pericytes into myofibroblasts (57). Thus, our findings indicate that the cellular and molecular interaction between immune cells and non-immune cells is crucial for the development of fibrotic responses in the lung during chronic allergic inflammation.

Various types of immune cells including effector Th2 cells, regulatory T cells, macrophages, and ILC2s contribute to the tissue homeostasis in different organs. In the liver, Amphiregulin is associated with liver regeneration and fibrotic responses (58). In the heart, Amphiregulin from Ly6C^lo^ macrophages is involved in the adaptive response to pressure overload (59). Moreover, recent research showed that Amphiregulin from Treg cells contributes to the suppression of neurotoxic astrogliosis in the brain (60). We await careful studies to elucidate the physiological and pathogenic roles of Amphiregulin in various tissues.

## Conclusion

We briefly reviewed the impact of the immune cell-epithelial/mesenchymal interaction on the pathology of chronic allergic inflammation. Tertiary lymphoid tissue, such as iBALT, is a key structure involved in the maintenance of memory T cells at mucosal sites. These ectopic lymphoid structures and the heterogeneity of memory-type pathogenic T (Tpath) cells may contribute to the induction of resistance to certain therapies that is observed in various immune-mediated inflammatory diseases. The dysregulation of type 2 immune responses via various stimuli among immune cells and epithelial/mesenchymal cells at mucosal sites induces many allergic inflammatory diseases, such as asthma. It will therefore become increasingly important to understand the precise roles played by immune cells and non-immune cells in overcoming intractable immune-mediated inflammatory diseases.

## Author Contributions

All authors listed have made a substantial, direct and intellectual contribution to the work, and approved it for publication.

### Conflict of Interest Statement

The authors declare that the research was conducted in the absence of any commercial or financial relationships that could be construed as a potential conflict of interest.
